# Spotlight on pro-inflammatory chemokines: regulators of cellular communication in cognitive impairment

**DOI:** 10.3389/fimmu.2024.1421076

**Published:** 2024-07-01

**Authors:** Chenxu Wang, Jiayi Wang, Zhichao Zhu, Jialing Hu, Yong Lin

**Affiliations:** ^1^ Department of Anesthesiology, The First Affiliated Hospital of Gannan Medical University, Ganzhou, China; ^2^ Department of Endocrinology and Metabolism, The Second Clinical Medical College of Nanchang University, Nanchang, China; ^3^ Department of Emergency Medicine, The Second Affiliated Hospital of Nanchang University, Jiangxi Medical College, Nanchang, China; ^4^ Ganzhou Key Laboratory of Anesthesia, The First Affiliated Hospital of GanNan Medical University, Ganzhou, China

**Keywords:** chemokines, cognitive impairment, T cell, neuroinflammation, neurodegenerative diseases

## Abstract

Cognitive impairment is a decline in people’s ability to think, learn, and remember, and so forth. Cognitive impairment is a global health challenge that affects the quality of life of thousands of people. The condition covers a wide range from mild cognitive impairment to severe dementia, which includes Alzheimer’s disease (AD) and Parkinson’s disease (PD), among others. While the etiology of cognitive impairment is diverse, the role of chemokines is increasingly evident, especially in the presence of chronic inflammation and neuroinflammation. Although inflammatory chemokines have been linked to cognitive impairment, cognitive impairment is usually multifactorial. Researchers are exploring the role of chemokines and other inflammatory mediators in cognitive dysfunction and trying to develop therapeutic strategies to mitigate their effects. The pathogenesis of cognitive disorders is very complex, their underlying causative mechanisms have not been clarified, and their treatment is always one of the challenges in the field of medicine. Therefore, exploring its pathogenesis and treatment has important socioeconomic value. Chemokines are a growing family of structurally and functionally related small (8–10 kDa) proteins, and there is growing evidence that pro-inflammatory chemokines are associated with many neurobiological processes that may be relevant to neurological disorders beyond their classical chemotactic function and play a crucial role in the pathogenesis and progression of cognitive disorders. In this paper, we review the roles and regulatory mechanisms of pro-inflammatory chemokines (CCL2, CCL3, CCL4, CCL5, CCL11, CCL20, and CXCL8) in cognitive impairment. We also discuss the intrinsic relationship between the two, hoping to provide some valuable references for the treatment of cognitive impairment.

## 1 Introduction

Neurocognitive disorders are a group of diseases in which the main clinical deficit is cognitive function. Dementia is considered to be a generic term for a wide range of neurocognitive disorders (1). There are many types of neurocognitive disorders, such as AD, PD, and multiple sclerosis (MS). They are characterized by a progressive deterioration in cognitive functioning and deteriorating physical, behavioral, mental, and emotional health (2). Studies have shown that cognitive disorders are very common among the elderly and the chances of developing cognitive disorders continue to increase with age. Suffering from such disorders is not only distressing for the patients themselves but also creates a huge financial burden on the family (3). Among them, AD is the most common type of dementia. Currently, nearly 47 million people around the world have been diagnosed with dementia. And this number is increasing due to the aging population (2). Previous studies have shown that patients with AD have elevated inflammatory markers in their blood. Inflammation affects the blood-brain barrier (BBB), which in turn leads to the development of AD (4). PD is the second most common neurological disorder after AD (5), with a global prevalence of more than 6 million people (6). Similar to AD, the prevalence of Parkinson’s increases with age (5). It has been investigated that it affects up to 2% of people over the age of 60 (7). In addition, findings show that men are more prone to the disease compared to women (6). It is characterized by a range of symptoms including tremors, rigidity, and dyskinesia (8). However, available experiments have confirmed that the effects of PD on patients are no longer limited to these aspects; it also diminishes the sense of smell and leads to depression, immune dysfunction, and even hallucinations (6, 9). MS also has a serious impact on people’s lives. MS is a chronic, autoimmune, inflammatory disease of the central nervous system (CNS) (10), which causes visual, cognitive, and sensory disturbances (11). MS also causes tremors to some extent (12). It is estimated that MS affects nearly 3 million people worldwide and most of them are less than 50 years old (11, 13). In a survey of gender, it was found that the number of women with MS is significantly greater than that of men (13). In terms of clinical manifestations, MS is mainly characterized by focal inflammation and degenerative lesions (14). Previous studies have shown that neuroinflammation has a very strong influence on cognitive impairment diseases. Chemokines play a very important role in neuroinflammation. Chemokines are a class of small proteins with the ability to attract and activate leukocytes, it is proven to play a crucial role in cognitive impairment. In this review, we will discuss the role of chemokines in neurocognitive disorders in detail.

## 2 Pro-inflammatory chemokine

### 2.1 Biological concepts and classification of chemokines

Chemokines are a class of small proteins (8–14 kDa) with the ability to attract and activate leukocytes, which can be classified into two groups according to their prominent functions: inflammatory chemokines and homeostatic chemokines (15–18). Some inflammatory chemokines have homogeneous functions, while others may be upregulated under certain “extraordinary” conditions (16). Chemokines are classified into four families based on the number and spacing of cysteine residues present at the N terminus. According to the systematic nomenclature, these families are named CXC (CXCL1–17), CC (CCL1–28), CX3C (CX3CL1), and C (XCL1/XCL2) (19–21), where C stands for cysteine and X for any amino acid residue (22). **Table 1** was mentioned in text but no corresponding table and caption provided. Please provide the table and caption.Each chemokine has its traditional name but under the new nomenclature introduced in 2000, chemokines are also considered as chemokine ligands, and each chemokine is referred to as CXCL, CCL, CX3CL1 or C (23). Chemokines exert their biological effects through the seven transmembrane structural domains of the G protein-coupled receptors present on the cell surface (19, 24), which are accordingly referred to as CXCR, CCR (1–10), CX3CR, and CR. Some chemokine receptors, such as CXCR2 and CCR1, bind multiple ligands, and many chemokines are also capable of binding to multiple receptors. While other chemokine receptors, such as CXCR4 and CXCR5, are specific for a single ligand (25, 26). Some chemokine receptors are not involved in cell migration or cell activation upon binding to ligands, but they can influence chemokine availability and function. These receptors are known as atypical chemokine receptors (ACKR 1–4) and are also important molecules involved in health and disease processes (27).

CXC chemokines have one amino acid residue in the middle of the first two cysteine residues (28). The genes encoding CXC chemokines are largely clustered on chromosome 4q12-q13 (23). The CXC chemokine family contains 17 members, such as GROα/MGSA/CXCL1, GROβ/CXCL2, and GROγ/CXCL3 (16).

The first two of the four cysteine residues of CC chemokines are adjacent to each other (29). Most of the genes encoding CC chemokines are located on chromosome 17q11.2 (23). The CC chemokine family contains 28 members, such as I-309/TCA-3/CCL1, MCP-1/CCL2, and MIP-1 α/CCL3 (16, 29).

The first two cysteines of the CX3C chemokine are separated by three amino acids and the gene encoding it is localized on human chromosome 16q (17, 30). The single member of the CX3C chemokine family is Fractalkine/CX3CL1 (21).

C chemokines have no neighboring cysteines (17). The C chemokine family contains two members, Lymphotactin/ATAC/SCM-1α/SCYC-1/XCL1 and SCM-1β/SCYC-2/XCL2 (21, 31).

### 2.2 Functions of pro-inflammatory chemokines

Inflammatory chemokines are involved in inflammation, tissue differentiation, hematopoiesis, and immune regulation (27). It is upregulated under inflammatory conditions and is mainly involved in leukocyte recruitment to inflamed tissues (16), directing leukocyte migration and promoting cell adhesion, invasion, mobilization, interaction with extracellular matrix, and survival (32). In addition, inflammatory chemokines direct the transport of phagocytosed leukocytes, activate leukocytes to produce superoxide anion, and release granular contents, thus enabling leukocytes to act as the first line of host defense against invading microorganisms (33). However, leukocyte accumulation and activation can also cause tissue damage, which can ultimately lead to inflammation, autoimmunity, and transplant rejection (32).

CXC chemokines recruit neutrophils, and lymphocytes and regulate angiogenesis. The CXC subfamily can be divided into ELR+ and ELR- CXC chemokines based on the presence or absence of a characteristic glutamate-leucine-arginine (ELR) motif near the N-terminus. Most ELR+ CXC chemokines directly induce endothelial cell activation to promote angiogenesis, whereas ELR- CXC chemokines inhibit angiogenesis (27, 34). CC chemokines have chemotactic effects on monocytes, lymphocytes, eosinophils, basophils, and dendritic cells (27, 28). CX3C chemokines are chemotactic for T lymphocytes and monocytes (22, 25). The chemokine structural domain of CX3CL1 fuses with mucin-like stalks, transmembrane regions, and cytoplasmic regions to form a cell adhesion receptor capable of blocking cells under physiological flow conditions (35). C chemokines are chemotactic for T lymphocytes, B lymphocytes, and natural killer cells (31).

### 2.3 Pro-inflammatory chemokines in disease

Inflammatory chemokines are key players in many disease processes including autoimmune diseases such as rheumatoid arthritis, atherosclerosis, asthma, and psoriasis, infectious diseases such as sepsis, HIV, and AIDS, ischemia/reperfusion injuries, cancers such as lung cancer, melanoma, and cognitively impaired disorders (32, 36–38). Cognitive impairment is the focus of attention in this review.

In the immune system, various pro-inflammatory chemokines produced by immunomodulatory cells or antigen-presenting guide the migration of these cells through the body (39). Some chemokines, such as CXCL4, CXCL12, CCL3, and XCL1, are involved in the development of B- and T-lymphocytes and play a crucial role in triggering the immune response. Receptors for chemokines also function as immune adjuvants and dendritic cells can recruit various T-cell subsets by specifically producing various chemokines (23, 40).

In the CNS, inflammatory chemokines go beyond their classical chemotactic function. Changes in the expression of inflammatory chemokines and their respective receptors are increasingly implicated in the pathogenesis of cognitively impaired diseases (e.g., AD, PD) (19). Its functions include neuromodulatory actions, neurotransmitter-like actions, regulation of the neuroendocrine axis, control of BBB permeability, regulation of neurogenesis, neuroprotection, neurotoxicity, and regulation of axonal germination and elongation (41). For example, the BBB restricts the entry of inflammatory leukocytes into the CNS, whereas inflammatory chemokines can disrupt the integrity of the BBB through the production of the matrix metalloproteinase MMP, which allows leukocytes to pass through the vessel wall, accumulate in the perivascular space, and then pass through the neuroglial cells to enter the parenchymal cells, triggering a destructive inflammatory response. Inflammatory chemokines also regulate cell proliferation, migration, and survival in the CNS (42). In a word, inflammatory chemokines and their receptors are very promising for research in cognitive disorders and could be important potential therapeutic targets.

## 3 The Role of major pro-inflammatory chemokines in cognitive impairment

### 3.1 CCL2

CCL2 is a member of the c-c family of pro-inflammatory chemokines, also known as monocyte chemotactic protein-1 (MCP-1). It mediates and enhances its biological effects by interacting with its G-protein coupled receptor, C-C chemokine receptor 2 (CCR2) (43). CCL2 is produced by numerous sources of CNS resident cells such as microglia, oligodendrocytes, and astrocytes (44). As a pro-inflammatory chemokine, it has significant chemotactic activity, and can effectively recruit and activate monocytes, such as macrophages and microglia, so that they can rapidly reach inflamed or damaged areas in response to lesions (45). CCL2 production pathways on astrocytes and microglia have similarities and differences. In the astrocyte pathway, astrocytes release large amounts of CCL2 when stimulated by cytokines (e.g. TNF-α) (46), which binds to the CCR2 receptor in microglial cells to activate the microglia into an M1-polarised state and induce their migration, thereby triggering the production of large amounts of inflammatory cytokines by microglia, which triggers neuroinflammation (47). In the microglia generation pathway, microglia, as the main immune cells, will directly produce CCL2 to recruit immune cells to clear the pathogens after encountering the pathogen stimulation, forming the neuroinflammatory effect (48). The initial purpose of the neuroinflammatory effect is to clear pathogens and maintain the stability of the nervous system. However, under certain pathological conditions, repeated activation of immune cells and repeated recruitment of chemokines create a vicious cycle that keeps the nervous system in a constant state of inflammation. Ultimately, this ongoing inflammatory process may lead to neuronal damage, triggering neurodegeneration that may result in neurodegenerative diseases associated with cognitive impairment. The onset of Mild Cognitive Impairment (MCI), a state of cognitive decline between normal aging and dementia, is associated with CCL2 levels. In subjects with MCI, cerebrospinal fluid and plasma levels of CCL2 are significantly elevated, and CCL2 may serve as a prognostic biomarker for the rate of cognitive decline in patients with MCI (49, 50).

This review on the mechanism of action of CCL2 in cognitive impairment disorders focused on AD. The main pathological features of AD are age spots for amyloid formation and neurofibrillary tangles (NFTs) composed of hyperphosphorylated tau proteins (50). In the generation of age spots, there are three main stages: shearing of Amyloid Precursor Protein (APP), misfolding and initial self-aggregation, and further accumulation. Aβ peptides are fragmented from precursor proteins by β-secretase and γ-secretase, and the cleaved fragments usually consist of 40 or 42 amino acids and therefore are named Aβ40 and Aβ42 (51). When Aβ peptides are formed in large quantities in the brain, misfolding and self-aggregation of proteins occurs, resulting in the formation of aggregates of varying structure and size. These aggregates can progressively form larger oligomers from monomers, which form amyloid fibrils together with further aggregation, which are deposited in the brain to form age spots. During the formation of senile plaques, the deposited amyloid fibers and the initially formed Aβ oligomers can induce the formation of chemokine CCL2, which can further activate immune cells such as astrocytes and microglia, prompting them to release inflammatory mediators, accelerating inflammatory reactions, leading to neuroinflammation and damage to neurons, and triggering neurodegenerative pathologies (52). While Aβ induces CCL2, CCL2 also plays an important role in the clearance of Aβ. In the early stages of AD development, microglia can mediate both inflammation and phagocytosis to degrade Aβ (53). Several studies have shown that a decrease in CCL2 leads to impaired phagocytosis of microglia, the lack of CCR2 receptors impairs microglia accumulation, thereby increasing Aβ accumulation, confirming the neuroprotective role of the CCL2-CCR2 pathway (54, 55). However, during the development of Aβ deposition, the formation of immunogens from Aβ deposition activates the immune action of immune cells, which release pro-inflammatory cytokines such as CCL2 to generate inflammation. Microglia are activated by inflammatory mediators through a vicious cycle of repeated stimulation, culminating in the dystrophic phenotype. Although microglia with dystrophic phenotype lose the mechanism of Aβ clearance, their pro-inflammatory effect still exists, and they continue to release CCL2 and other chemokines to enhance the inflammatory response, and the malignant inflammatory response severely damages neurons and synaptic function, leading to the development of AD (53, 55). Although CCL2 can mediate Aβ elimination, studies have shown that overexpression of CCL2 may lead to the generation of Aβ oligomers (56). Oligomers are highly neurotoxic and in addition to causing neuroinflammation, oligomers can affect synaptic plasticity, oxidative stress, and interference with intracellular signaling. It has been shown that binding of Aβ oligomers to α-Amino-3-hydroxy-5-methyl-4-isoxazole propionic acid receptor (AMPAR) decreases the activity of AMPAR and interferes with the long-term potentiation (LTP), resulting in cognitive dysfunction (57). In terms of oxidative stress, the combination of Aβ oligomers to N-methyl-D-aspartate receptors (NMDAR) can elevate Ca2+ concentration in neuronal cells, leading to increased intracellular oxidative stress, dendritic spine deletion, and even neuronal cell death (58, 59). Meanwhile, overexpression of CCL2 can also lead to the overproduction of IL-6, which, in addition to acting as a chemokine in the inflammatory response, can also stimulate the production of precursor protein in neurons *in vitro*, which also supports the fact that CCL2 mediates the production of Aβ oligomers (60).

The production and elimination of Tau protein, another neuro marker of AD, which is a microtubule-associated protein that polymerizes microtubule proteins into microtubules and maintains the neuronal structure, is also closely related to CCL2 (61). Tau proteins undergo a variety of post-translational modifications, such as glycosylation, but phosphorylation is predominantly the mainstay of post-translational modifications (62). Nearly 30 sites can be phosphorylated in normal tau proteins, but overphosphorylation causes tau proteins to detach from microtubules and form NFTs (61, 62). Over-phosphorylated Tau proteins activate immune cells such as microglia, promote the release of pro-inflammatory cytokines, activate inflammatory responses, and damage neurons (63). Meanwhile, pathological Tau proteins can impair mitochondrial function, and NFTs lead to neuronal synapse loss and axonal transport disorders (61). Joly-Amado et al. showed that CCL2 overexpression can cause tau phosphorylation, causing related diseases (60). Vaz et al. showed that increased secretion of CCL2 can lead to overphosphorylation of tau, which promotes NFT generation (64). All these findings demonstrate the mediating nature of CCL2 for tau phosphorylation and the facilitating nature of disease progression.

**Figure 1:**
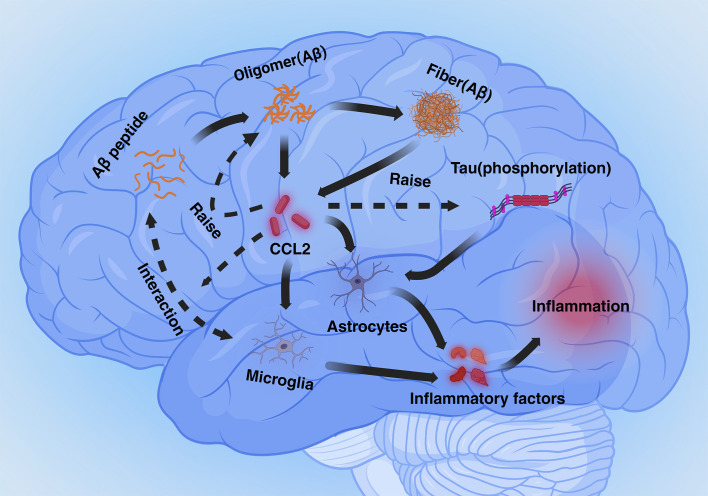
Mechanisms mediated by CCL2 in Alzheimer’s disease (Inflammation section). Aβ and phosphorylated Tau proteins cause astrocyte- and microglia-mediated neuroinflammation via CCL2. Excess CCL2 promotes Aβ oligomer generation as well as phosphorylation of Tau proteins, and CCL2 also plays a role in phagocytosis of Aβ by microglia.

CCL2 plays a dual role in two important physiological markers of AD. On the one hand deficiency of the CCL2-CCR2 pathway reduces Aβ elimination by microglia and promotes Aβ accumulation. On the other hand, overexpression of CCL2 causes accumulation of Aβ oligomers, APP generation, and hyperphosphorylation of Tau. The dual role of CCL2 may be closely related to the dual role of microglia in processing the markers, with microglia being in a neuroprotective phenotype early in the pathogenesis and becoming neurotoxic in the later stages. Although there is no research data to elucidate the mechanism by which CCL2 leads to this dual role for the time being, differences in the expression of CCL2 content and temporal differences between early and late pathogenesis are of interest to study this dual role (**Figure 1**).

**Figure 2:**
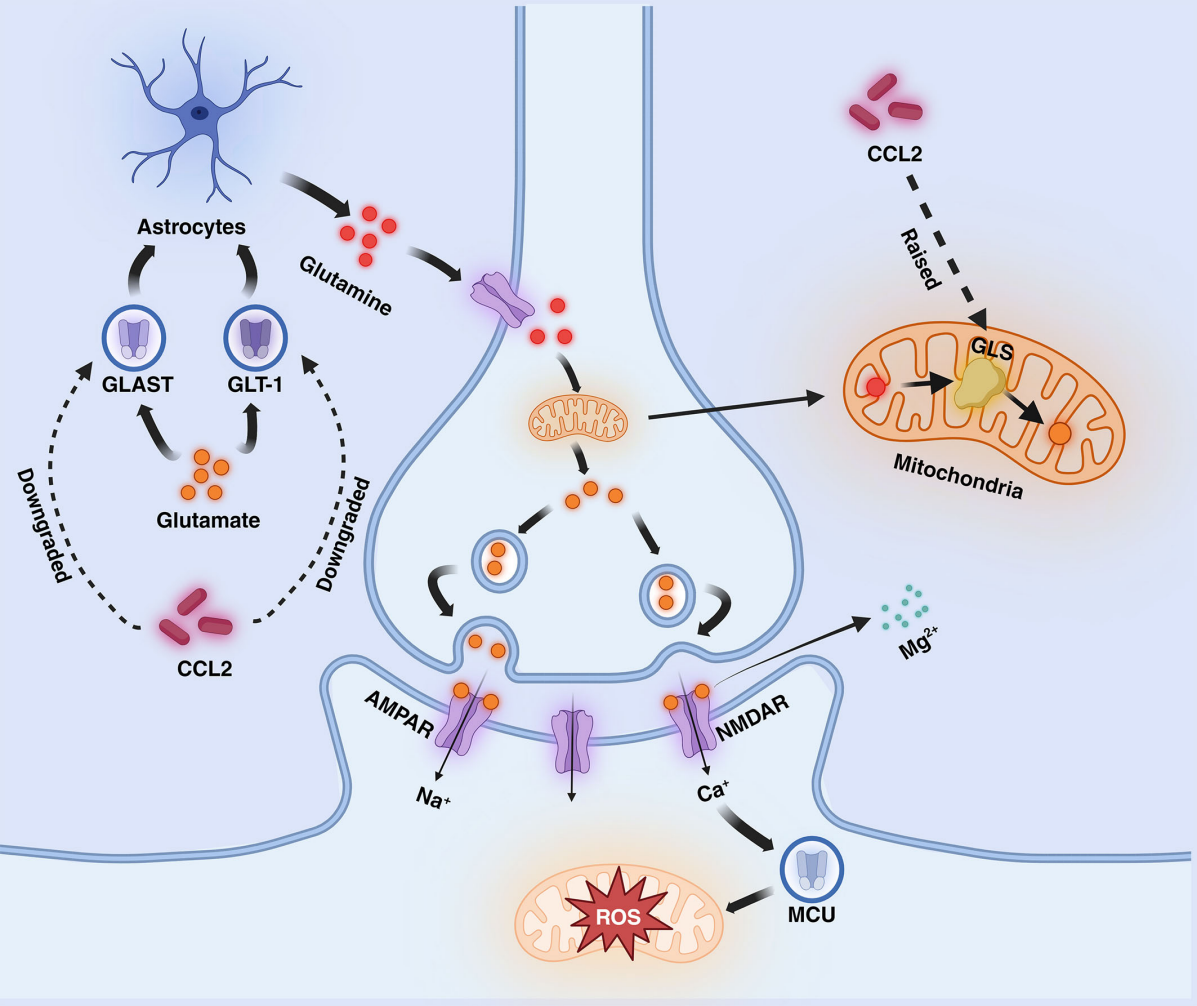
Mechanisms mediated by CCL2 in AD (excitotoxicity section). CCL2 dramatically increases glutamate levels by decreasing glutamate transport by GLT-1 and GLAST and increasing glutamate synthesis by GLS in astrocytes, leading to NMDAR hyperactivation, increased calcium influx into the mitochondria, and the generation of large amounts of ROS. Fatty acid oxidation (FAO) and oxidative phosphorylation (OXPHOS) also increase the generation of ROS. AMPAR, α-Amino-3-hydroxy-5-methyl-4-isoxazole propionic acid receptor; NMDAR, N-methyl-D-aspartate receptors; ETC, electron transport chain; GLT-1, Glutamate transporter-1; GLAST, Glial Glutamate Transporter; GLS, Glutaminase; MCU, unidirectional transport protein.

Excitotoxicity is the process by which nerve cells are damaged by overstimulation, which usually manifests itself as elevated glutamate (Glu) concentration, leading to the overactivation of receptors on the posterior membrane (65). Glu is an excitatory neurotransmitter that plays an important role in inter-synaptic messaging, synaptic remodeling, and neuroplasticity and binds to a variety of receptors, including glutamate-binding calcium-permeable ionotropic receptors known as NMDAR and AMPAR (66). In the nervous system, Glu, which is more abundant in the synaptic gap, can be recycled by the Glu transporters GLAST and GLT-1 on astrocytes and synthesized into glutamine (Gln), which is transported to the presynapseand and catalyzed by a deamination reaction catalyzed by glutaminase (GLS) in the mitochondrion, which catalyzes the formation of Glu (67). It has been shown that CCL2 increases the expression of GLS and downregulates the expression of GLAST and GLT-1 (68). When CCL2 levels are increased, this leads to reduced functional levels of excitatory amino acid transporter proteins and reduced Gln conversion in astrocytes, while enhanced GLS expression promotes Glu conversion, ultimately leading to a substantial increase in Glu levels in the synaptic gap. activation of AMPAR with Glu results in a sustained influx of Na+ into the cell, leading to depolarization of the posterior membrane and the generation of an action potential, eliminating the blockage of NMDAR channels by Mg2+ in the resting state, then Ca2+ is free to enter the cell via NMDAR (65). High levels of Glu cause over-activation of NMDAR, resulting in a large influx of calcium into neurons. The neurons can take up a large amount of calcium through the mitochondrial calcium unidirectional transport protein (MCU) and slowly release it back into the cytoplasm, which can buffer the change of calcium level and maintain ATP balance (69). However, when calcium levels are elevated, persistently high levels of cytoplasmic calcium lead to mitochondrial calcium overload, which causes depolarization of the mitochondrial membrane and the production of large amounts of reactive oxygen species (ROS), which induces oxidative stress and enhances mitochondrial autophagic clearance, leading to abnormal mitochondrial function and ultimately to cell death (66, 70, 71). In addition to calcium overload, Fatty Acid Oxidation (FAO) and Oxidative Phosphorylation (OXPHOS) also have a large impact on ROS production. Intracellularly, fatty acids are oxidatively catabolized via the FAO pathway to acetyl coenzyme A, which then passes through the tricarboxylic acid cycle (citric acid cycle) to produce the reduced coenzymes NADH and FADH2. These reduced coenzymes are utilized by the process of oxidative phosphorylation within the mitochondria, releasing their electrons through the electron transport chain (ETC), ultimately converting ADP to ATP (72). However, when the efficiency of electron transfer in the electron transport chain decreases during oxidation, ROS are generated from incompletely reduced oxygen molecules, especially at complex I and complex III (73, 74). The increase in ROS continuously neutralizes the antioxidant enzymes, and the enzyme activity decreases as a result of overwork, leading to a lack of antioxidant capacity and accelerated promotion of oxidative stress. (**Figure 2**).

### 3.2 CCL3

CCL3 is a member of the CC subfamily of pro-inflammatory chemokines known as macrophage inflammatory proteins 1-alpha (75–77), which have been shown in several studies to be produced by monocytes/macrophages, lymphocytes, and neutrophils, as well as by immune cells such as basophils, mast cells, fibroblasts, and dendritic cells (75, 77, 78). CCL3 has been found to bind to a variety of cell surface receptors (CCR1 (75–77, 79), CCR3 (75), CCR5 (75–77, 79), and CCR4), exerting multiple biological effects. In the inflammatory response mechanism, CCL3 can promote the secretion of other pro-inflammatory cytokines by binding to CCR1, CCR4, and CCR5 receptors (76), and it also interacts with CCR1 and CCR5 to recruit a variety of immune cells to sites of inflammation to promote an inflammatory response (77). Neuroinflammation is one of the main causes of cognitive disorders and diseases, and current research confirms that CCL3 can be involved in neuroinflammation (79), suggesting that CCL3 may contribute to the generation of neurological disorders, such as AD. A 2017 experiment found that increased expression of CCL3 prompted glial cells, monocytes, and lymphocytes to aggregate in a hypoxic mouse model. Scientists hypothesized that this could be linked to neuroinflammatory mechanisms in the pathogenesis of AD. In addition, CCL3, which is located in microglia, astrocytes, and perivascular macrophages, is upregulated in the CNS during AD, which also demonstrates the relevance of CCL3 to the pathogenesis of AD (80, 81). Hwang and his co-workers have found that cell death in patients with AD is associated with a decrease in CCR5, which leads to increased Aβ deposition. The Aβ deposition, which in turn leads to memory impairment. We currently speculate that the decrease in CCR5 may be associated with an increase in CCL3 and CCL4 (81).

### 3.3 CCL4

CCL4 is also a part of the CC subfamily of pro-inflammatory chemokines, which is known as macrophage inflammatory protein-1β (82, 83). On a smaller scale, CCL4 has a molecular weight of 7.8 kDa, its gene is located on chromosome 17, and 92 amino acid precursors constitute a normal CCL4 protein; on a larger scale, CCL4 exists in the form of a symmetric homodimer in the shape of an elongated cylinder. It was found that CCL4 is mainly secreted by immune cells, fibroblasts, endothelial cells, and epithelial cells (84). Among the immune cells, the main ones include monocytes, B-lymphocytes, and T-lymphocytes (85). Similar to CCL3, CCL4 plays a vital role in inflammation and immune modulation (86). CCL4 is secreted by several of these cells when triggered by specific signals and attracts macrophages, natural killer cells, monocytes, etc. to participate in the immune response (87). In the immune response, CCL4 activates acute centrophilic inflammation and induces the synthesis and release of inflammatory cytokines such as IL-1, IL-6, and TNF-a from fibroblasts and macrophages (88). CCL4 is involved in various physiological processes such as bone marrow activation, calcium mobilization, etc. Here we focus on the effects of CCL4 in cognitive disorders diseases. Some research data suggest that CCL4 levels are elevated in the blood of schizophrenic patients, and that increased CCL4 leads to BBB dysfunction. Thus, CCL4 may be involved in the pathogenesis of schizophrenia (89). CCL4 also plays a very important role in AD. Inflammation is a prominent feature in the development of Alzheimer’s patients (90), and Aβ deposition contributes to neuroinflammation. And CCL4 plays a tremendous role in the inflammatory response (91). AD occurs mainly in older people. Compared to younger people, miR-125b is less abundant in older adults. It has been found that CCL4 expression is regulated by miR-125b, and a decrease in miR-125b is associated with a significant increase in CCL4 (85). A 2014 experiment demonstrated that the expression of CCL4 and its receptor CCR5 was increased in Alzheimer’s patients and virtually absent in normal brains (90). Increased CCL4 also adversely affects the ability of astrocytes to remove Aβ, leading to aβ deposition, Aβ-induced neuroinflammation, and AD (90).

### 3.4 CCL5

CCL5, C-C chemokine motif ligand V, a normal T cell expressed and secreted factor known to be regulated upon activation (92), is also called RANTES (93). It belongs to the C-C motif chemokine family. In human chromosomes, the gene encoding CCL5 is located on chromosome 17q11.2-q12. It has been found that CCL5 can be produced by a variety of cells, including T cells, macrophages, eosinophils, synovial fibroblasts, and endothelial cells. Of these, CCL5 is mainly derived from T cells and macrophages (94). CCL5 controls the migration of memory B cells, monocytes, macrophages, and eosinophils to the CNS, so it can play a part in a variety of neuroinflammatory-like diseases (95). Examples include AD, MS, etc. A 2018 study showed significantly elevated levels of CCL5 expression in astrocytes from rats with spinal cord injury. CCL5 can promote the migration of microglia in spinal cord-injured rats, and the migration of microglia is involved in neuropathogenesis. This demonstrates that CCL5 aggravates neuropathological changes after CNS injury (96). Of the multiple CCL5 receptors, the more important are CCR1 and CCR5. CCL5 contributes to BBB damage associated with multiple neurologic diseases upon binding to CCR1 (97). The CCL5/CCR5 axis plays a major role in the pathogenesis of AD. In the early pre-pathogenesis period, microglia and astrocytes are mobilized by Aβ deposition, initiating the Aβ clearance mechanism. CCL5 increases NO secretion from activated microglia, decreases IL-10 and IGF-1 production, and reduces their ability to clear Aβ deposits. This in turn exacerbates the induction of neuroinflammation by Aβ precipitation and triggers the disease (98). In addition, CCL5 complementarily inhibited the MAPK/CREB signaling pathway and exacerbated synapse formation defects. In experiments, rats in the CCL5 knockdown group were found to be partially rescued from synaptic degeneration. miR-324–5p regulates the expression of CCL5, and reduced miR-324–5P expression and elevated CCL5 expression were found in the brains of senescent mice, suggesting that the miR-324–5P-CCL5 axis contributes to the loss of synapses during aging (99). This in turn exacerbates neurocognitive deficits.

### 3.5 CCL11

CCL11, also known as eosinophil chemotactic protein (eotaxin-1), is a member of the eosinophil chemokine family. It is an eosinophil chemokine involved in innate immunity (100). Cytokines from T-helper (Th)-2 cells, such as IL-13 (interleukin-13), IL-10 (interleukin-10), and IL-4 (interleukin-4), induce the production of CCL11. CCL11 is a product of eosinophils, T cells, B cells, fibroblasts, epithelial cells, endothelial cells, macrophages, chondrocytes, and microglia (101), and is transported from the blood to the brain via the BBB (102, 103). The main receptor for CCL11 is CCR3 with higher affinity than other CCL11 receptors like CCR2 and CCR5 (100, 104). Microglia, astrocytes, and neurons all express CCR3, suggesting that CCL11 can affect multiple targets in the CNS (102).

CCL11 may have biphasic neuroprotective and neurotoxic effects depending on factors such as concentration, anatomical location, and pathophysiological context (103). CCL11 plays a crucial role in the development of cognitive dysfunction and is an immune marker of aging and accelerated aging. Its highly proliferative properties are associated with cognitive deficits in executive function, situated memory, and semantic memory. Accordingly, CCL11 has been called endogenous cognitive deterioration chemokine (ECDC) or accelerated brain aging chemokine (ABAC) (105, 106). Elevated levels of CCL11 have been demonstrated in neuroinflammatory diseases such as MS, psychiatric disorders such as schizophrenia, and neurodegenerative diseases such as PD and AD (106–108).

CCL11 can cause cognitive impairment by inhibiting neurogenesis (103, 106), reducing synaptic density (106), and inducing neuronal cytotoxicity leading to neuronal damage and death. Activated neuroglial cells can trigger neuroinflammation (109). Activated astrocytes or BBB crossover sources release CCL11, while activated microglia express CCR3 (105). CCL11 binding to microglia-expressed CCR3 up-regulates nicotinamide adenine dinucleotide phosphate oxidase 1 (NOX-1), promotes the production of ROS by microglia, which induces inflammation and neuronal cytotoxicity leading to neuronal cell death (110, 111).

The primary pathogenesis of PD is neuronal degeneration in the midbrain substantia nigra, leading to neurological damage and loss of brain cells due to neuroinflammation and loss of neuronal connectivity (112). In an animal model, the production of pro-inflammatory factors and CD4+/CD8+ T-cell infiltration in the substantia nigra was reduced by administration of anti-CCL11 neutralizing antibody, prevented ameliorated motor symptoms and nigrostriatal neurodegeneration in PD mice (113), indicating a potential mechanism of CCL11 in PD.

CCL11 has been recognized as a possible risk factor for the development of AD. It is elevated in the serum of AD patients but not altered in the cerebrospinal fluid (CSF) (114, 115). However, less is known about its involvement in the pathogenesis of AD (111). The main pathogenesis of AD is the degeneration of neurons in the hippocampal gyrus and the cerebral cortex, with the hippocampal region playing a crucial role in learning and memory processes. Neuroinflammation causes hippocampal atrophy (116), which may play an important role in the development of AD (109, 114). A study showed that injecting plasma from aging mice or CCL11 into young mice impaired learning and memory and decreased adult neurogenesis, suggesting that CCL11 is involved in the decline of hippocampal function during aging (116). CCR3, which is present in microglia, is more highly expressed in reactive microglia in AD patients. Knockdown of CCR3 in mice reduces synaptic loss and improves memory deficits and spatial learning, further suggesting that CCL11 may increase the risk of AD (111).

T cells, as important immune cells, play an important role in CCL11-mediated neuroinflammation. The migration and adhesion of T lymphocytes across the microvascular endothelium is an important event in their recruitment to sites of inflammation, and their infiltration from the periphery into the brain is thought to be an important component in many neurodegenerative diseases involving dysfunction of the BBB. The Th1 cytokine IL-2 and the Th2 cytokine IL-4 induce the expression of CCR3 by T lymphocytes, which in turn attract T lymphocytes through CCR3. In addition, CCL11, in combination with IL-2 and IL-4, increases the expression of adhesion molecules such as ICAM-1 and various integrins (CD29, CD49a, and CD49b) on T lymphocytes, thus promoting the adhesion and aggregation of T lymphocytes. The specific cAMP-dependent protein kinase inhibitor H-89 selectively blocked CCL11-induced T cell adhesion, indicating that CCL11 activates T cells through a specific cAMP signaling pathway. These results indicate that CCL11 is an important activator of T lymphocytes, stimulating the directed migration, adhesion, aggregation, and recruitment of T lymphocytes. The activation of CCL11 by T lymphocytes is therefore an important factor in disease prevention. Inhibition of peripheral T lymphocytes expressing CCR3 during aging reduces the infiltration of these T lymphocytes across the BBB, thus attenuating neuroinflammation, which suggests that CCR3 inhibition has vast therapeutic potential for a range of neuroinflammatory diseases (117–119).

These suggest a role for CCL11 in the pathological mechanism of cognitive impairment. Resveratrol, Glucocorticoids, minocycline, CCR3 antagonists, and anti-CCL11 antibodies have been shown to attenuate CCL11 production (111).

### 3.6 CCL20

CCL20, namely cysteine motif chemokine ligand 20, also known as macrophage inflammatory protein-3α (MIP-3α), liver activation-regulated chemokine (LARC), and Exodus-1, is a small-molecule protein of approximately 8 kDa released primarily from neurons, astrocytes, and microglial cells of the CNS, and is encoded by the SCYA20 gene located on human chromosome 2 (120–123). CCL20 has a unique receptor, the C-C chemokine receptor type 6 (CCR6) (121, 124, 125), and CCR6 is preferentially expressed by Th17 cells (123, 125).

CCL20 is chemotactic for dendritic cells (DCs), T cells, and B cells (120, 124). It is associated with tissue inflammation, infectious diseases, and several types of cancers (120, 125, 126), and may be involved in brain nerve damage and neuroinflammation (127). It has been shown that CCL20 plays an important role in neurodegenerative changes following trauma, spinal cord injury, and cerebral ischemia in rodents (120). Neutralization of CCL20 or knockdown of CCR6 resulted in decreased microglia activation and reduced neuroinflammation, suggesting that neurodegenerative and inflammatory effects are at least partially mediated by CCL20 (120, 123). Brain Treg cells inhibit the neurotoxic proliferation of astrocytes and their infiltration into the brain is controlled by CCL20. CCL20 can also recruit Treg cells through FOXO1/CEBPB/NF-kappaB signals (128–130). These studies further demonstrate the role of CCL20 in mediating inflammation, particularly in the field of immune cells. However, the role of CCL20 in cognitive disorders is unclear.

### 3.7 CXCL8

CXCL8 or interleukin 8 (IL-8), namely chemokine (C-X-C motif) ligand 8, is an inflammation-promoting chemokine and belongs to the CXC group of chemokines (131, 132). CXCL8 consists of a proximal promoter region, four exons, and three introns, and specific genes on chromosome 4q13-q21 encode CXCL8 (133, 134). CXCL8 is produced by endothelial cells, macrophages, adult fibroblasts, epithelial cells, lymphocytes, and airway smooth muscle cells (134, 135). Cytokines from T lymphocytes stimulate epithelial cells to produce CXCL8 (136). Moreover, CXCL8 is virtually undetectable under physiological conditions but can be quickly induced by inflammatory cytokines, such as TNF-α and IL-1b, or by bacterial and viral products (132, 137). The G protein-coupled serpentine receptors CXCR1 and CXCR2 are the most frequently studied receptors for CXCL8. CXCL8 binding to the receptors can help activate inflammatory PKB, MAPK, and PKC signaling pathways (137–139). CXCR1 and CXCR2 are referred to as IL-8 receptor A (RA) and IL-8 receptor B (RB). They belong to the GPCR family, have seven transmembrane structural domains, and are expressed in mast cells, monocytes, granulocytes, and NK cells (134, 140).

The main functions of CXCL8 are the recruitment and activation of neutrophils (131, 132, 137), the promotion of monocyte and macrophage proliferation, the increase of the MMP-2 and MMP-9, endothelial cell proliferation, angiogenesis (107, 135, 139), and the enhancement of oxidative metabolism and ROS which cause oxidative stress (131).

In the blood and CSF of AD patients, CXCL8 levels have been demonstrated to be significantly increased (141–143). The function of CXCL8 in the pathogenesis of AD remains unclear, but IL-6, IL-1b, TNF-α, and COX-2 levels increase when culturing human microglia with Aβ in the presence of CXCL8, suggesting that CXCL8 may be responsible for bringing the activated microglia to AD-injured brain regions as an alternative recruitment mechanism, which is important for the increase of Aβ pro-inflammatory responses (144, 145). The CXCL8 receptor has been localized to dystrophic neuronal synapses, further suggesting that CXCL8 may mediate the interaction of microglia with neurons leading to neuronal injury (141, 146). CXCL8 also affects the induction of the pro-apoptotic protein and the cell cycle protein cyclin D1, as well as the release of MMP-2 and MMP-9, which may act as important mediators of neuronal death in AD (133). In addition, CXCL8 levels were lower in patients with moderate AD than in those with severe AD, demonstrating that CXCL8 levels are correlated with AD severity significantly and may serve as a biomarker for monitoring AD progression (145).

**Figure 3:**
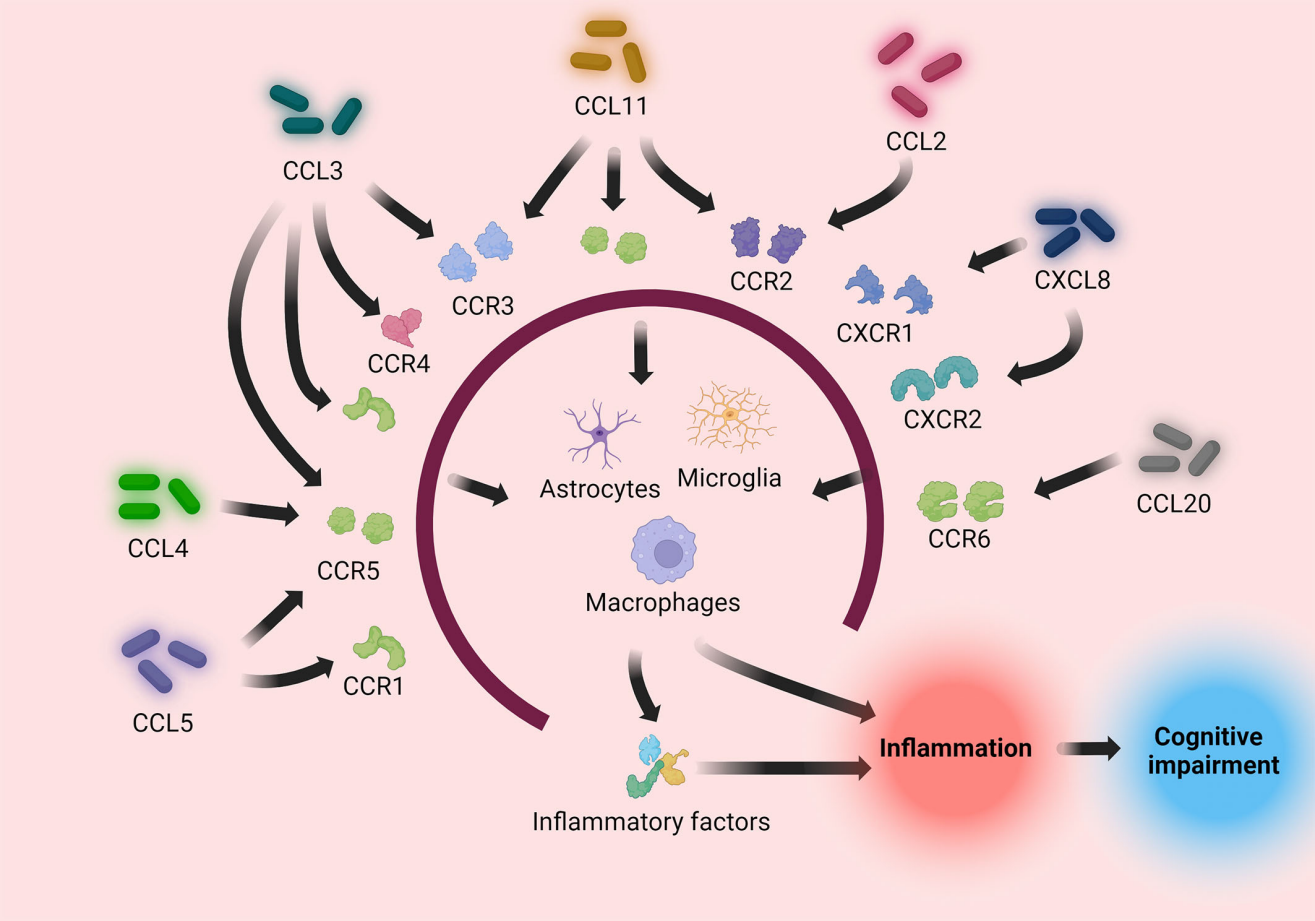
Pro-inflammatory chemokines (CCL2, CCL3, CCL4, CCL5, CCL11, CCL20, CXCL8) induce immune cells to directly or indirectly process pathogens by binding to their respective receptors, causing an inflammatory response. The inflammatory response causes neuronal injury, causing cognitive impairment.

Nevertheless, some experiments suggest that CXCL8 may be neuroprotective rather than neurotoxic (147). Although treatment of human neurons with CXCL8 alone had no significant effect on their survival or apoptosis, CXCL8 inhibited Aβ-induced neuronal apoptosis and increased neuronal production of brain-derived neurotrophic factor (BDNF), which may protect neurons through paracrine or autocrine secretion. Consequently, CXCL8 may play a protective role in the pathogenesis of AD (146) (**Table 1**; **Figure 3**).

**Table 1:** Receptor for chemokine ligands and the role of chemokine ligand in cognitive impairment.

Chemokines lignads	Receptors	The role of chemokine ligands
CCL2	CCR2	Inflammation and Excitotoxicity
CCL3	CCR1	Inflammation
	CCR3	
	CCR4	
	CCR5	
CCL4	CCR5	Inflammation
CCL5	CCR1	Inflammation
	CCR5	
CCL11	CCR2	Inflammation and Oxidative stress
	CCR3	
	CCR5	
CCL20	CCR6	Inflammation
CXCL8	CXCR1	Inflammation,Induction of neurotoxins and Pro-apoptotic proteins
	CXCR2	

## 4 Treatment strategies

Current therapies for cognitive disorders regarding the chemokine perspective mainly work by inhibiting chemokine-induced inflammatory responses and oxidative stress. In the neuroinflammatory response, chemokines not only recruit immune cells and mediate the release of pro-inflammatory cytokines, but also activate microglia, astrocytes, and other neuroglial cells to enhance the inflammatory response. Long et al. demonstrated that naringenin reduced the mRNA expression of IL-1β and IL-6, two interleukins, in the CCL2 group, and that the levels of IL-1β and IL-6, important pro-inflammatory cytokines in the body, were reduced. The decrease in the content of IL-6, an important pro-inflammatory cytokine in the body, proves that the drug has anti-inflammatory effects on ccl2-mediated neuroinflammation (148). Liao et al. also found that the expression of IL-1β and IL-6 decreased in the hippocampus of rats pretreated with Tanshinone IIA, which exerted an obvious anti-inflammatory effect (149). Regarding anti-inflammatory drugs, non-steroidal anti-inflammatory drugs occupy a certain position. NSAIDs such as diclofenac, ibuprofen, and indomethacin can limit the development of inflammation and thus limit the occurrence of neurodegenerative diseases such as AD. Ibuprofen can delay the onset of the inflammatory response and reduce damage to neurons by limiting the activation of microglia and astrocytes from becoming pro-inflammatory phenotypes (150). Some other NSAIDs may also reduce neuronal damage by affecting the spatial conformation of Aβ and thus its concentration in the brain (151). Thalidomide, as a Glu derivative, is commonly used as an anti-tumor and anti-inflammatory analgesic, and in the nervous system, it can inhibit the activation of microglia and astrocytes, thus limiting the release of pro-inflammatory cytokines from glial cells, lowering the concentration of pro-inflammatory cytokines in the nervous system, and reducing the inflammatory response (152, 153). During the drug action, thalidomide can better cross the BBB, which is more pro-inflammatory to the CNS compared with steroidal anti-inflammatory drugs (154). Reboxetine, as an antidepressant drug, has also been studied to reduce the damage to the brain caused by the massive production of Aβ protein (155).

From an oxidative stress perspective, antioxidant mechanisms exist in the cell, and two major antioxidant systems, SOD and GSH-Px, exist to maintain the balance between oxidation and antioxidation (148). When the organism is injured, ROS in the mitochondria increases greatly, and the balance between oxidation and antioxidants is imbalanced, which damages the mitochondria, leading to abnormal mitochondrial function, which in turn leads to neuronal damage. Pananx notoginseng saponins, while having anti-inflammatory effects, can significantly increase the expression of the SOD enzyme system to attenuate the effects of chemokines on mitochondrial oxidative stress (68). As an excitatory amino acid receptor antagonist, memantine can inhibit the excitotoxicity of Glu and reduce the generation of ROS by acting on the Glu receptor NMDAR, and its low affinity makes it inhibit the toxicity without interfering with the normal synaptic transmission, which reduces the interference with the normal physiological effects (156).

Current treatments focus on the physiological response to chemokine receptor binding, such as glial cell activation and excitotoxicity due to Glu overload. Therapies that interfere with the binding pathways of chemokines and receptors are rare, and if the binding of both can be directly restricted, it may be possible to address the root cause of the inflammation. Most of the chemokines and receptors that have been identified so far are many-to-one in combination, with one receptor receiving different chemokines. If treatments could be more inclined to inhibit receptor binding than to limit the number of chemokines, it would also reduce the difficulty and complexity of the study.

## 5 Conclusion

The family of inflammatory chemokines causes neuroinflammation mainly through various mechanisms such as inducing neuroglia and promoting the production of inflammatory mediators. Widespread and persistent neuroinflammation results in neuronal damage that affects neuronal function and promotes the development of neurodegenerative diseases and cognitive impairment. This review describes some of the more important inflammatory chemokines and their pathogenic mechanisms in cognitive disorders. However, the pathogenic mechanism of some chemokines is still unclear, and further research is needed.

## Author contributions

CW: Writing – original draft. JW: Writing – original draft. ZZ: Writing – original draft. JH: Writing – review & editing, Supervision, Conceptualization. YL: Writing – review & editing, Supervision, Conceptualization.
